# Applications of OralCDx ® methodology in the diagnosis of oral leukoplakia

**DOI:** 10.4317/medoral.17275

**Published:** 2011-07-15

**Authors:** Flavio Seijas-Naya, Tamara García-Carnicero, Pilar Gándara-Vila, Elena Couso-Folgueiras, Mario Pérez-Sayáns, Rafael Gándara-Vila, Abel García-García, José-Manuel Gándara-Rey

**Affiliations:** 1 Dental surgeon. Student of the Master of Oral Medicine, Oral Surgery and Implantology; Faculty of Dentistry of the University of Santiago de Compostela; 2 Dental surgeon. Student of the Master of Oral Medicine, Oral Surgery and Implantology; Faculty of Dentistry of the University of Santiago de Compostela; 3 Dental surgeon. Professor of the Master of Oral Medicine, Oral Surgery and Implantology; Faculty of Dentistry of the University of Santiago de Compostela; 4 Biologist Anatomical Pathology Technician at the Anatomical Pathology Unit of the Santiago de Compostela Teaching Hospital; 5 PhD in Dental Surgery Associate Professor of the Master of Oral Medicine, Oral Surgery and Implantology; Faculty of Dentistry of the University of Santiago de Compostela; 6 Dentist. Collaborator of the Master of Oral Medicine, Oral Surgery and Implantology; Faculty of Dentistry of the University of Santiago de Compostela; 7 Maxillofacial surgeon Professor of Oral Surgery Director of the Master of Oral Medicine, Oral Surgery and Implantology; Faculty of Dentistry of the University of Santiago de Compostela. Head of the Maxillofacial Surgery Unit of the Santiago de Compostela Teaching Hospital; 8 Stomatologist Professor of Oral Medicine Director of the Master of Oral Medicine, Oral Surgery and Implantology; Faculty of Dentistry of the University of Santiago de Compostela

## Abstract

Objective: We aim to evaluate the effectiveness of the brush biopsy technique using OralCDx ® (OralScan Laboratories Inc., Suffern, NY) as a new method for early diagnosis and control of a “potentially malignant disorder” such as oral leukoplakia. Design of the study: We performed a study in which samples were taken using OralCDx ® on 24 patients who visited the Master of Oral Medicine, Oral Surgery and Implantology of the University of Santiago de Compostela between February 2009 and May 2010. These patients presented clinical and histological lesions that were consistent with oral leukoplakia. We evaluated the relationship between the keratinization degree of the lesions and cell representation; the diagnosis obtained through OralCDx ® and biopsies; and sensitivity, specificity, positive predictive value (PPV) and negative predictive value (NPV). Results: 50% of patients were men and 50% women with an average age of 62.38 years. The Kappa coefficient relating keratinization of lesions and cell representation was 0.33, the OralCDx ® - biopsy diagnostic rate reached a Kappa value of 0.66, recording 72.7%,sensitivity and 92.3% specificity, PPV was 88.8%, while NPV reached 80%. Conclusions: cytology sampling with OralCDx ® showed high sensitivity and specificity values, which make it a good tool for monitoring oral leukoplakia, but nowadays the most reliable method that allows us to confirm the exact diagnosis of the lesions and their anatomical and pathological characteristics still is conventional biopsy using a surgical scalpel.

** Key words:** OralCDx®, brush biopsy, oral leukoplakia.

## Introduction

 Oropharyngeal cancer is the sixth most common malignancy worldwide (1). Among these cases, oral cancer accounts for approximately 650,000 new cases each year (2). In Western countries it represents 2-4% of all malignancies (3). 40% of head and neck tumors are Oral Squamous Cell Carcinomas (OSCC) (2), amounting to 90% of the total of all those affecting the oral cavity (4). Early detection of oral cancer in early stages affects the chances of survival significantly (1) thus improving the quality of life of these patients after undergoing treatment (3). 

Survival of OSCC patients has not improved over the last 30 years (5); the 5-year survival rate reaches 80% in cases detected at the initial stage, 40% in cases of regional involvement, and less than 20% in cases with distant metastasis (2). The delay in diagnosis is due to the fact that patients do not seek oral care for an unusual oral situation which is coupled with health professionals’ lack of knowledge about these lesions (4). 

The aforementioned terms, such as “precancerous lesion”, “precancerous condition” or “intraepithelial neoplasia” have been replaced in May 2005 by the World Health Organization in collaboration with the Center for Oral Cancer and Precancer in the United Kingdom with the term ‘Potentially Malignant Disorders”, which included oral leukoplakia among other illnesses (6). Early detection of these potentially malignant disorders is key in preventing an accumulation of alterations, such as dysplasia, increasing their risk of a malignant transformation (7). 

Dentists therefore play an important role in early detection of malignant and premalignant conditions and should examine all patients at risk. (8,9). Biopsy is still the most accepted technique to reliably detect the nature of oral mucosal lesions, but it is a bloody test that implies undergoing surgery and the resulting technical limitations for some professionals, in addition to the procedure’s psychological implications for patients (5). Due to this, it is important to develop new diagnostic techniques and instruments that can be used routinely (8). 

Oral exfoliative cytology is the microscopic study and interpretation of the characteristics of oral mucosal cells that are shed (naturally or artificially), by means of a simple, non-invasive and relatively painless technique that is well accepted by patients (4). Its use had been disregarded due to its low sensitivity level that is patent in the high number of false negatives (4), possibly due to inadequate sampling, technical errors and a poor design of sampling instruments (10). The implementation of “Cytobrush” in the 90’s has revived interest in oral exfoliative cytology since it increases the number of collected cells (11). Newer techniques such “brush biopsies” can assess cellular benignity or malignancy by computer-assisted analysis (5). Recently, we have begun to use exfoliative cytology as more sophisticated methods of DNA and molecular analyses, which leads to improve its quality and reliability as a cancer diagnostic technique (4). 

This study aims to assess efficacy of a new brush biopsy technique, OralCDx ® (OralScan Laboratories Inc., Suffern, NY), as a new method for early diagnosis and monitoring a “potentially malignant disorder” such as oral leukoplakia.

## Material and Methods

 We conducted a study (a controlled clinical trial), using samples obtained through OralCDx ® on 24 patients who visited the Master of Oral Medicine, Oral Surgery and Implantology of the University of Santiago de Compostela, referred by the SERGAS (Servizo Galego de Saúde - Galician Public Healthcare System), between February 2009 and May 2010 who showed clinical and histological lesions that were consistent with oral leukoplakia in different clinical forms.

 -Selected lesions

Oral leukoplakia is a potentially malignant disorder with a prevalence of 0.2% to 3.6% of oral cavity white lesions, which amount to 24.8% of oral lesions (12). Warnakulasuriya et al. defined this lesion in May 2005, as a white plaque with an increasing questionable oral cancer risk after excluding other known diseases and disorders that do not increase the risk. Sometimes, red areas appear alongside the white ones; this is called erythroleukoplakia. Leukoplakia often presents a clear correlation with smoking habits, but there are some idiopathic cases (6). The annual average of malignant transformation is 1%. (12). 

 -Method

OralCDx ® (OralScan Laboratories Inc., Suffern, NY), is a brush biopsy method with computer assisted sample analysis. It obtains cells from the three cell layers of the epithelium of the oral mucosa for a correct analysis (13). 

The Kit contains a brush cytology sampling tool, a pre-coded glass slide, two pre-coded forms, two sachets of polyethylene alcohol fixative, a container for the sample holder and an envelope to send the samples (9). For sampling, we followed the steps indicated by the manufacturer: filling forms (a copy to be sent out and a copy for the dentist); brush sampling by performing 10 to 20 lateral or frontal rotations, in a representative area (never on an ulcer) until the area turns reddish or a light dotted hemorrhage; we then transferred the sample on to the pre-coded slide, placing the fixative to avoid contamination of the sample; the sample is then left to dry for 15 minutes and placed in the container and the envelope for shipment. In the laboratory the sample is stained with Papanicolaou tint (13); then it is scanned and analyzed microscopically using a computer with an image database containing different degrees of abnormal cell morphology (14). The program is capable of detecting two abnormal cells among thousands of normal cells (13). A cytopathologist reviews and interprets the data (14). The results are classified as atypical (cellular changes of uncertain diagnosis), positive for dysplasia or carcinoma, negative (normal cells) and inappropriate (incomplete transepithelial sample) (15). With all this data a report is issued and sent to the dentist.

The OralCDx® brush biopsy technique is a simple, minimally invasive, relatively inexpensive, painless and has shown greater psychological acceptance by patients (3,13). With it, we can obtain complete transepithelial samples to perform adequate analysis of the lesion (2,13). No special training is required for its use thus favoring its acceptance among professionals (5,16). Local anesthesia is rarely necessary except in cases of ulcerated lesions in which its use is indicated, since sampling of these areas often results in greater discomfort for the patient (13). 

Besides the different variables appearing on the form (color, location, symptoms, etc.) the dentist responsible for the cytological samples subjectively assessed the keratinization degree of the lesions. 

We decided to consider as low-keratinized lesions, regardless of the oral cavity area in which they were located, those lesions that had a similar color to that of healthy oral mucosa. Moderately keratinized lesions were those having a whitish appearance but that were located in areas of the oral cavity showing little or no keratinization (e.g. buccal mucosa) and highly keratinized lesions were those showing a high degree of keratinization and were located in keratinized areas (e.g. hard palate).

All patients had a sample taken with a surgical scalpel 3 weeks prior or after sampling with the OralCDx ® kit to compare our results.

 -Inclusion-Exclusion criteria

All patients were informed about the procedure they were to undergo, they were asked to sign a consent form authorizing sampling. They also had to authorize the performance of a conventional biopsy using a surgical scalpel.

We excluded patients who did not sign the consent form for both samples; those who had undergone treatment before the lesion and those whose lesions had a different diagnosis from leukoplakia. All samples were taken by the same dentist and analyzed by the same pathologist.

The development of this study was approved by the Ethics Committee of the Faculty of Medicine and Dentistry, University of Santiago de Compostela.

We used the computer program SPSS 15.0 for Windows (Inc., Chicago, USA) to analyze the different variables of our study, using contingency tables to calculate the Kappa index and assess the correlation between test results using OralCDx ® and conventional biopsy and the keratinization degree and cell representation. Calculations of sensitivity, specificity, positive predictive value (PPV) and negative predictive value (NPV) were performed using STATAtm software 10.1 for Windows.

## Results

 Of the 24 patients included in the study, 12 (50%) were men and 12 (50%) women. We took a single sample from each patient. Local anesthesia was not required in any of the cases. Their age ranged between 40 and 82 years, with an average age of 62.38 + / - 12.14 years. 19 patients (79.16%) were initially diagnosed with oral leukoplakia and 5 with erythroleukoplakia (20.83%). The most frequent location of lesions in our study was the lateral border of tongue, appearing in 8 cases (33.3%), which was followed by buccal mucosa in 4 cases (16.7%). In our cases, 11 (45.8%) of the lesions showed a flat appearance and 13 (54.2%) were verrucose. 15 (62.5%) patients were smokers or had a history of smoking, while 9 of these (60%) drank alcohol regularly.

Concerning the degree of keratinization, we found 6 cases (25%) of highly keratinized lesions, 14 (58.3%) were moderately keratinized and 4 lesions (16.7%) were slightly keratinized.  Table 1 shows all data concerning the characteristics of the lesions.

**Table 1:**
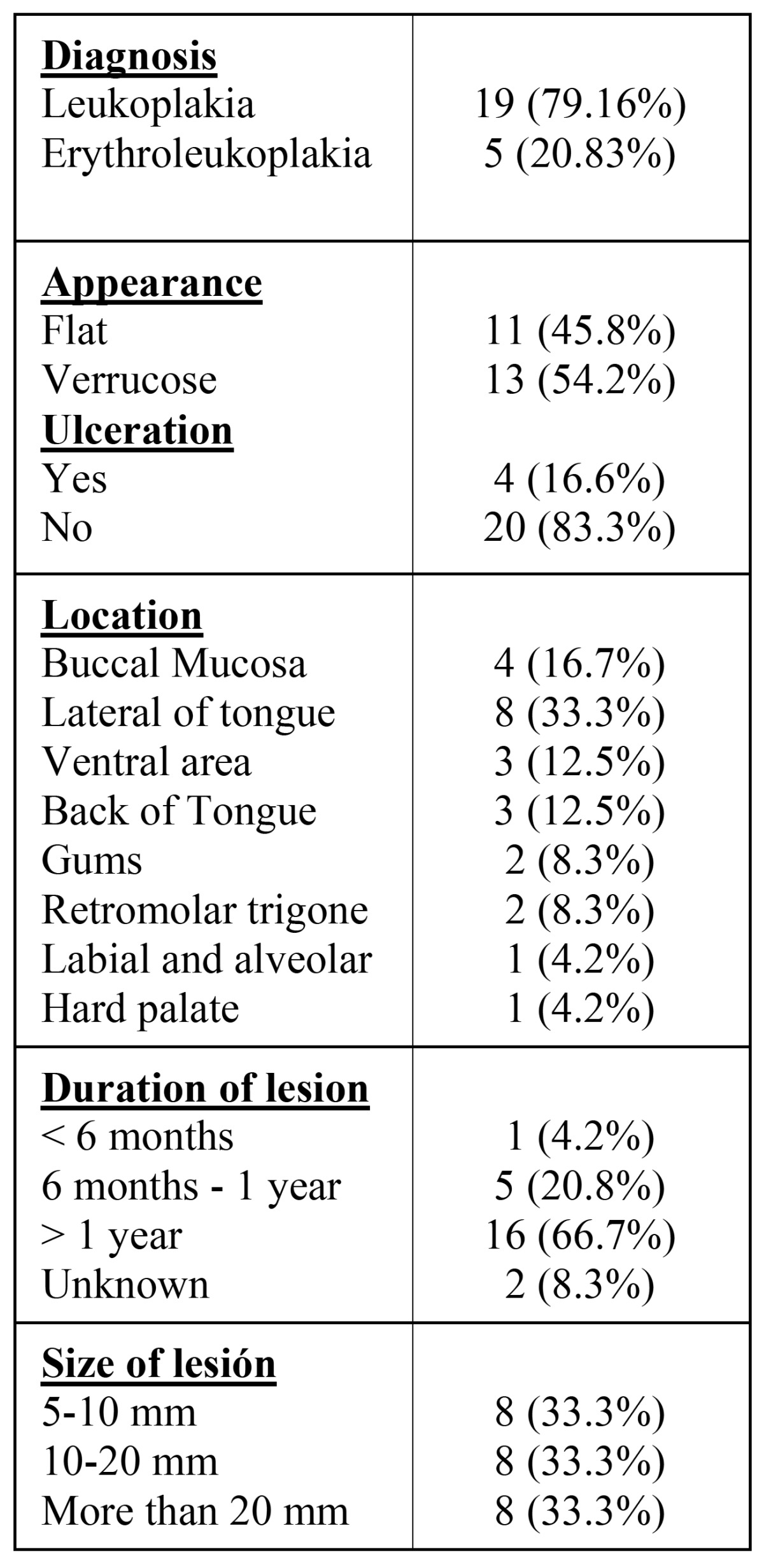
Clinical characteristics of lesions.

As regards to the OralCDx ® technique, in 16 (66.7%) of cases we obtained cells from all 3 layers of the epithelium (superficial, intermediate and basal), while in 4 (16.7%) cases we found intermediate and superficial cells and in 4 (16.7%) we only obtained superficial cells.

In the analyses reports we found that the tests were ne-gative (no cellular alterations) in 15 cases (62.5%) and that there was atypia in the remaining 9 cases (37.5%). Local anesthesia during the sampling procedure was not necessary in any of the cases in our study.

The results of surgical scalpel biopsies were classified as negative (without epithelial alteration) in a total of 13 cases (54.2%); atypical (mild-moderate dysplasia) in 11 cases (45.8%).

By correlating these two variables, test results using OralCDx ® and conventional biopsies, and taking into account all the samples, we obtained values of 72.7% sensitivity and 92.3% specificity. PPV was 88.8% (95% CI 50.6-99.4%), while NPV was 80% (95% CI 51.3-94.6%). Upon calculating correlation between the results obtained with OralCDx ® and biopsies we obtained a Kappa value of 0.66, which showed good correlation.

Finally, we analyzed the keratinization degree in relation to the difficulty of obtaining a complete sample and we observed that in the total number of cases of low-keratinized lesions (4 samples) we obtained a complete sample; in 4 (28.5%) of the 14 cases with moderately keratinized lesions we only obtained superficial and intermediate cells; while in 4 of 6 (66.6%) cases of highly-keratinized lesions we only managed to obtain an incomplete sample. The Kappa value in this case was 0.33 thus we recorded a low correlation, however it was very close to the correct values (0.4).

## Discussion

 In our study we found that the lateral border of the tongue was the most frequent location of the lesions appearing in 8 cases (33.3%), followed by buccal mucosa in 4 cases (16.7%). Meanwhile other authors state that the most common location is the buccal mucosa with rates varying between 31 and 22%, followed by the lateral border of tongue (15-22%). (1,13,16). This difference may be due to the small size of our sample compared with that of other authors (1,13,16).

Of all our patients, 15 (62.5%) were smokers or had a history of smoking which indicates the relationship between the occurrence of these injuries and smoking. 

In our study and in those by other authors we found that samples were taken mainly in predominantly white lesions with percentages ranging from 40-65% (1,13,16,17).

As regards to the cellularity obtained by sampling with the OralCDx ® kit; in 8 cases (33.3%) cells we did not obtain cells from the 3 layers of the epithelium; 4 of which (50%) were taken at the beginning of our study, which is normal due to our initial unfamiliarity with the technique. (13,15). The average of incorrect samples usually ranges between 2% and 6% (15). This relatively low percentage of incomplete samples was due to the rigid design of the brush for adequate sampling (13,18). Upon evaluating the difficulty in obtaining complete samples in relation to the lesion’s keratinization degree, we noted that in 4 (66.6%) of 6 highly-keratinized lesions we could not obtain cells from the 3 layers of the epithelium, while in 4 (16.7%) of 14 cases of moderately-keratinized lesions we failed to obtain a complete sample, recording a Kappa value of 0.33, which was very close to matching correct correlation values for this variable (0.4). Many authors have indicated that a high keratinization degree in some lesions can be contradictory to the use of this technique since it hinders us from securing sufficient cellularity to perform adequate analysis (8,11,13,15). We could then consider, in these cases, taking a sample using a scalpel which would allow for a correct analysis of these lesions.

Correlating the results of OralCDx ® and conventional biopsy we recorded a Kappa value of 0.66, which indicated a high concordance level. The sensitivity value for this test was 72.7% and 92.3% specificity. Both va-lues are close to those found in other publications which range between 70-100% for sensitivity and between 90-100% specificity (11,13,15-17). We found one false positive (11.1%). 

This relatively low sensitivity may be due to the fact that we found 3 (20%) false negatives. One of these cases showed a high degree of dysplasia. It is important to note that basal layer cell representation layer in this specific patient was insufficient. In fact, if we were to eliminate all 8 cases in which cell representation was incomplete we would obtain a sensitivity of 87.5% while the specificity would remain the same. PPV was 88.8% and NPV 80%, values that somewhat corroborate the reliability of our test. These values vary in other publications, ranging between 38-88% for PPV and 60-100% in the case of NPV (14).

In most studies, a biopsy was taken only in patients who showed atypical cytology results. This can cause errors in the studies of these authors, in terms of sensitivity, specificity, PPV and NPV calculations (18). Authors such as Scheifele et al., indicate that they performed biopsies only in cases of atypia, with a delay of 75 to 292 days, which may cause major histopathologic changes in the lesion during that lapse of time. (15). In our study no more than 21 days passed between sampling with one technique or the other.

The main problem we found was that with the OralCDx ® kit we failed to establish diagnoses that allowed us to identify the lesion, given that it only allows us to perform an assessment of the cellular representation, thus it may be considered as a method to monitor lesions. Nowadays, to obtain definitive certainty of diagnosis it is still necessary to use a conventional scalpel biopsy (2). Another problem we observed was scarceness of pathologists who are specialized in the analysis of these samples, as has been previously noted by other authors (13). Often, reference laboratories have a long delay in issuing results with deadlines ranging from 30 to 120 days. 

Another problem noted by some authors is sampling with OralCDx ® in an inflammatory pathology such as lichen planus or pemphigus, since these conditions often result in the existence of a greater number of false positives, as a result of cell morphology which has been altered by inflammation. (8,13).

Multiple researches are underway to try to prove the future applications of this methodology in cytomorfometry, molecular analysis and DNA collection and analysis from liquid-based cytology. (3-5,8,11,15,19,20).

Our conclusion is that, although cytological sampling with OralCDx ® (OralScan Laboratories Inc., Suffern, NY), presents high sensitivity and specificity values that make it a good tool for monitoring oral leukoplakia, nowadays, the most reliable method to confirm the exact diagnosis of lesions and their histopathologic characteristics biopsy is still conventional biopsy by surgical scalpel.
